# Zero Dollar Drug Copay program improves antidiabetic medication adherence and medication use patterns among Blue Cross and Blue Shield of Louisiana members with diabetes in Louisiana

**DOI:** 10.1136/bmjdrc-2025-005146

**Published:** 2026-01-20

**Authors:** Tiange Tang, Charles Stoecker, Debra Winberg, Mingyan Cong, Miao Liu, Elizabeth Nauman, Yun Shen, Gang Hu, Hui Shao, Jian Li, Alessandra N Bazzano, Eboni Price-Haywood, Brice Labruzzo Mohundro, Jason Ouyang, Mollie Carby, Lizheng Shi

**Affiliations:** 1 Health Management and Policy, Tulane University School of Public Health and Tropical Medicine, New Orleans, Louisiana, USA; 2 Blue Cross and Blue Shield of Louisiana, Baton Rouge, Louisiana, USA; 3 Louisiana Public Health Institute, New Orleans, Louisiana, USA; 4 LSU Pennington Biomedical Research Center, Baton Rouge, Louisiana, USA; 5 Hubert Department of Global Health, Rollins School of Public Health, Atlanta, Georgia, USA; 6 UNC-Chapel Hill, Chapel Hill, North Carolina, USA; 7 Ochsner Health System, New Orleans, Louisiana, USA

**Keywords:** Medication Adherence, Program Evaluation

## Abstract

**Objective:**

Blue Cross and Blue Shield of Louisiana (BCBSLA) launched a zero-dollar co-pay (ZDC) pharmacy benefit on July 1, 2020, to reduce cost-related barriers to diabetes medications. This study evaluated the program’s effect on antidiabetic medication adherence and use patterns.

****Research Design and** Methods:**

We conducted a retrospective cohort study using BCBSLA medical and pharmacy claims from 2019–2021. The study included 7,603 continuously enrolled members with diabetes: 3,045 fully insured members with ZDC coverage (ZDC group) and 4,558 administrative-services-only members without ZDC coverage (control group). Follow-up was July 1, 2020, to December 31, 2021. Outcomes included monthly proportion of days covered (PDC), drug counts, and monthly medication use. We applied propensity score odds weights and estimated weighted difference-in-differences models with individual and time fixed effects, adjusting for demographics, comorbidities, healthcare utilization, and spending. Subgroup analyses examined pre-ZDC users, pre-ZDC non-users, and complex users.

**Results:**

Mean age was 48.8 (SD 12.5) years in the ZDC group and 52.9 (SD 11.3) years in controls; 57.4% and 55.6% were female, respectively. The ZDC program increased PDC by 4.4 percentage points (p<0.001), monthly medication use by 6.2 percentage points (p<0.001), and drug counts by 0.090 (p<0.001). For ZDC-eligible medications, increases were 5.4 percentage points for PDC, 7.6 percentage points for monthly use, and 0.074 for drug counts (all p<0.001). Improvements were observed among pre-ZDC users and complex users, but not among pre-ZDC non-users.

**Conclusion:**

A zero-dollar co-pay pharmacy benefit improved antidiabetic medication adherence and increased medication use among BCBSLA members with diabetes.

**WHAT IS ALREADY KNOWN ON THIS TOPIC**

**WHAT THIS STUDY ADDS**

- This paper underscores the potential effect of VBID on enhancing medication adherence. Specifically, our research focuses on patients with diabetes and provides a unique perspective on studying the effect of VBID based on medication use patterns.

**HOW THIS STUDY MIGHT AFFECT RESEARCH, PRACTICE OR POLICY**

- This paper informs healthcare decision-making by demonstrating how VBID can effectively improve medication adherence, ultimately contributing to better health outcomes and more efficient use of healthcare resources.

## Introduction

 Type 2 diabetes (T2D) is a major health concern in the USA and the state of Louisiana (LA). In 2022, more than 500,000 people (10.9%) in LA had diagnosed T2D, and more than 6% of the state population were at the pre-diabetes stage.^1^ T2D costs the state of LA about US$6.7 billion per year, with US$4.5 billion being direct medical costs.^1^

Non-adherence to antidiabetic medication plays a major role in the development of diabetic complications, increased healthcare utilization, productivity losses, and increased economic burden.^2^,^4^ Every 10% increase in non-adherence to metformin and statins is associated with a 0.14% increase in hemoglobin A1c (HbA1c) and a 4.9 mg/dL increase in low-density lipoprotein cholesterol.^5 6^ Approximately 50% of the global diabetes population has failed to reach optimal glycemic control due to poor medication adherence.^5 6^ Hence, improving medication adherence is crucial to diabetes management.

The factors associated with medication non-adherence among patients with T2D include, but are not limited to, financial hardship, lack of insurance, lower age, lower education level, female sex, and high comorbidity burden.^7 8^ Among all the factors, financial hardship plays a fundamental role, as most of the other contributing factors are related to insufficient economic power. Prior research showed that near-poor people with higher deductible health plans were more likely to experience financial hardships and less likely to adhere to prescribed medications.^9^ In addition, a systematic review reported that the change in the copayment was statistically significantly related to medication adherence and diabetes management inversely. For every US$10 increase in the copayment, the medication possession ratio decreases by 6%, and for every US$5 increase in the copayment, the HbA1c increases by 0.1%.^10^

In order to improve medication adherence, alleviate financial burden, and enhance clinical outcomes such as glycemic control among patients with T2D, Blue Cross Blue Shield in Louisiana (BCBSLA) initiated a Zero Dollar Copay (ZDC) pharmacy benefit for its fully insured members starting on July 1, 2020. The ZDC program provides a wide range of drug classes and eliminates the copays for antidiabetic medications, most of which are generics. This study evaluates whether the ZDC program improved medication adherence and medication use patterns among patients with T2D.

## Research design and methods

### Study design

This is a retrospective ecologic study.

### Data source

The claims data used for this study were provided by BCBSLA. BCBSLA’s claims data encompass a broad range of information, such as demographic details, enrollment information, medical claims, pharmacy claims, diagnosis and procedure codes, dates of service, information about healthcare providers and facilities, laboratory test results, referrals to other specialists, as well as financial and other billing codes.

### Study period

On July 1, 2020 (the index date), the ZDC pharmacy benefit became available for fully insured BCBSLA members. This study ranges from January 1, 2019 to December 31, 2021, which consists of an 18-month baseline period and an 18-month follow-up period (July 1, 2020, to December 31, 2021).

### Eligibility criteria

Members were required to be at least 18 years old at baseline and to be continuously enrolled with BCBSLA and have copay pharmacy benefits for the 3-year study period. We excluded members who had elected to participate in pilot iterations of the ZDC program, those residing outside the state of LA, individuals with type 1 diabetes, and members enrolled in high-deductible health plans or Medicare supplemental plans.

### Patient enrollment in the ZDC program

Members who were insured through firms that elected to use administrative services only (ASO) were not eligible for the ZDC program. In ASO agreements, a firm assumes the financial risk of the health insurance pool, but contracts with a health insurance company to manage the claims. This contrasts with traditionally administered plans (fully insured), where the health insurance company assumes the financial risk of the health insurance pool in addition to the administrative duties. All members enrolled in firms that used BCBSLA as a traditional administrator (fully insured) were automatically enrolled in the ZDC program if they had at least one of the following five conditions: diabetes, hypertension, heart disease, lung disease, or mental illness, in addition to meeting the aforementioned eligibility criteria. Self-selection into the program was not permitted. Aside from receiving the ZDC pharmacy benefit, no other differences were introduced between the two groups, apart from the inherent distinctions between fully insured and ASO health plans.

### Diabetes patient selection and study cohort

BCBSLA members with T2D were identified based on the presence of at least one International Classification of Diseases, 10th Revision code E11 in medical claims or a Generic Product Identifier (GPI) 2-digit major class code 27 in pharmacy claims. Fully insured BCBSLA beneficiaries with T2D comprised the ZDC group, and members with the BCBSLA ASO plan who did not have the ZDC benefit served as the control group for this study.

### Outcomes

The outcomes of this study include monthly proportion of days covered (PDC), measured by the number of days covered by a prescription within a month; monthly drug count, measured by the number of medications used; and monthly medication use, measured by the proportion of members using antidiabetic medication each month. Study outcomes were categorized based on medications’ eligibility for the ZDC program, with separate reports on all prescribed medications and those specifically eligible for ZDC.

### PDC calculation

To accurately estimate medication adherence, we applied a days of supply shifting method, aligning each fill to begin only after the previous supply was expected to run out, thereby avoiding overlap within the same medication. After shifting individual prescriptions, we aggregated records across different antidiabetic medications to construct continuous periods of medication coverage. This approach prevents double-counting overlapping fills and enables consistent calculation of the PDC (online supplemental appendix figure 1, page 16).

### Statistical analysis

Two-way fixed effects difference-in-difference (DID) regressions with weights by odds of propensity scores were performed in this study. Members in the ZDC group were assigned a weight of 1, while those in the control group were weighted by the odds of their propensity scores. Data from the first 3 months (January 2019 to March 2019) was excluded from regression analysis due to insufficient pharmacy claims of late 2018 (online supplemental appendix figure 5, page 17), however, we do keep the first 3 months of data for patients with T2D identification. Counts and percentages were reported for categorical variables, and means and SD were reported for continuous variables.

The regressions of study outcomes were weighted by odds of propensity scores and adjusted for age (continuous and categorical), chronic conditions, healthcare utilizations, urban–rural residence, medication spending, and diabetes complications severity index. We also adjusted for plan-type-specific linear time trends by estimating plan-type-specific linear time trends of the outcome variable based on preperiod data and then detrending over the full study period. The SEs of the coefficients were clustered at the individual level to account for potential serial correlations over time. Event studies and F-tests were performed before running the DID regression to test the preparallel trend assumption.

### Subgroup analysis

We further explored heterogeneous treatment effects across subgroups, defined solely based on baseline use of ZDC-eligible medications, which include all generic antidiabetic medications and some brand-name medications covered by BCBSLA (online supplemental appendix figure 6, page 18). Specifically, members with at least one claim for a ZDC-eligible antidiabetic medication during the baseline period were classified as pre-ZDC users. Those with no such claims were defined as pre-ZDC non-users. Complex users were defined as individuals who used both ZDC-eligible and non-eligible antidiabetic medications during the baseline period. These subgroups included both fully insured and ASO members. Within each subgroup, we further distinguished between adherent and non-adherent members. An adherent member was defined as having a baseline PDC of 0.8 or higher. Statistical significance was considered to be p<0.05. All analyses were performed using Stata/SE V.16.1.

## Results

The unweighted and weighted descriptive statistics of baseline demographic, clinical, and medication spending covariates are summarized in table 1. After applying weight to both groups, the baseline characteristics became comparable, with all standardized mean differences being smaller than 10%. Among the 7,603 members, 3,045 of them were eligible for the ZDC program. The average ages of individuals in the ZDC and control groups were 48.78 years (SD=12.45) and 52.93 years (SD=11.27), respectively. Among the population, 57.37% in the ZDC group and 55.55% in the control group were female. Additionally, 77.04% of individuals in the ZDC group and 80.87% in the control group lived in urban areas. Hypertension was the most prevalent chronic condition in both groups.

**Table 1:** Baseline covariates balance, all members, monthly PDC of all medications (n=7,603)

Variables	Non-weighted group monthly average			Weighted group mean monthly average		
	ZDC (n=3,045)	Control (n=4,558)	SMD	ZDC (n=3,045)	Control (n=3,039)	SMD
Age	48.78 (12.45)	52.93 (11.27)	−0.35	48.78 (12.45)	48.74 (12.52)	0.00
Age (≤45)	1,103 (36.22)	1,047 (22.97)	0.29	1,103 (36.22)	1,112 (36.6)	−0.01
Age (46–64)	1,750 (57.47)	3,032 (66.52)	−0.19	1,750 (57.47)	1,735 (57.08)	0.01
Age (≥65)	192 (6.31)	479 (10.51)	−0.15	192 (6.31)	192 (6.32)	0.00
Sex (women)	1,747 (57.37)	2,532 (55.55)	0.04	1,747 (57.37)	1,732 (57)	0.01
COVID-19	55 (1.81)	60 (1.32)	0.04	55 (1.81)	55 (1.82)	0.00
Anxiety	576 (18.92)	938 (20.58)	−0.04	576 (18.92)	581 (19.13)	−0.01
Cancer	284 (9.33)	517 (11.34)	−0.07	284 (9.33)	287 (9.44)	0.00
CHF	56 (1.84)	177 (3.88)	−0.12	56 (1.84)	60 (1.97)	−0.01
CAD	205 (6.73)	527 (11.56)	−0.17	205 (6.73)	210 (6.9)	−0.01
CKD	123 (4.04)	302 (6.63)	−0.12	123 (4.04)	128 (4.23)	−0.01
COPD	57 (1.87)	153 (3.36)	−0.09	57 (1.87)	57 (1.87)	0.00
ESRD	13 (0.43)	43 (0.94)	−0.06	13 (0.43)	14 (0.48)	−0.01
Hypertension	1,565 (51.4)	3,369 (73.91)	−0.48	1,565 (51.4)	1,576 (51.87)	−0.01
Osteoarthritis	384 (12.61)	770 (16.89)	−0.12	384 (12.61)	386 (12.71)	0.00
SAD	125 (4.11)	187 (4.1)	0.00	125 (4.11)	127 (4.19)	0.00
Urban	2,346 (77.04)	3,686 (80.87)	−0.09	2,346 (77.04)	2,344 (77.14)	0.00
Brand AA	376.25 (953.87)	460.67 (1583.3)	−0.06	376.25 (953.87)	428.79 (2845.56)	−0.02
Generic AA	59.08 (107.75)	87.09 (345.77)	−0.11	59.08 (107.75)	59.77 (97.28)	−0.01
IA	0.01 (0.03)	0.01 (0.03)	−0.05	0.01 (0.03)	0.01 (0.03)	−0.02
OP surgery	0.04 (0.12)	0.05 (0.11)	−0.09	0.04 (0.12)	0.04 (0.1)	0.00
PCP visit	0.18 (0.18)	0.22 (0.2)	−0.21	0.18 (0.18)	0.19 (0.17)	0.00
SO visit	0.53 (0.77)	0.59 (0.84)	−0.08	0.53 (0.77)	0.54 (0.94)	−0.01
Office visit	0.71 (0.81)	0.82 (0.89)	−0.12	0.71 (0.81)	0.72 (0.97)	−0.01
UC visit	0.03 (0.06)	0.04 (0.08)	−0.09	0.03 (0.06)	0.03 (0.06)	0.01
ER visit	0.02 (0.05)	0.02 (0.06)	−0.12	0.02 (0.05)	0.02 (0.05)	0.00
DCSI score	1.42 (2.4)	2.17 (2.76)	−0.29	1.42 (2.4)	1.44 (2.4)	−0.01
Drug counts	0.92 (0.81)	1.23 (0.94)	−0.35	0.92 (0.81)	0.94 (0.78)	−0.02
Preperiod monthly PDC	0.56 (0.36)	0.67 (0.34)	−0.32	0.56 (0.36)	0.57 (0.36)	−0.04

Table displays counts and percentages for categorical variables and means and SD for continuous variables. PDC was not included in the probit regression that predicted ZDC status, it is displayed to assessment balance across ZDC and control groups.

AA, allowed amount; CAD, coronary artery disease; CHF, congestive heart failure; CKD, chronic kidney disease; COPD, chronic obstructive pulmonary disease; DCSI, Diabetes Complication Severity Index; ER, emergency room; ESRD, end-stage renal disease; IA, inpatient admission; OP, outpatient surgery; PCP, primary care physician; PDC, proportion of days covered; SAD, substance abuse disorder; SMD, standardized mean difference; SO, specialty office; UC, urgent care; ZDC, Zero Dollar Copay.

**Figure 1:**
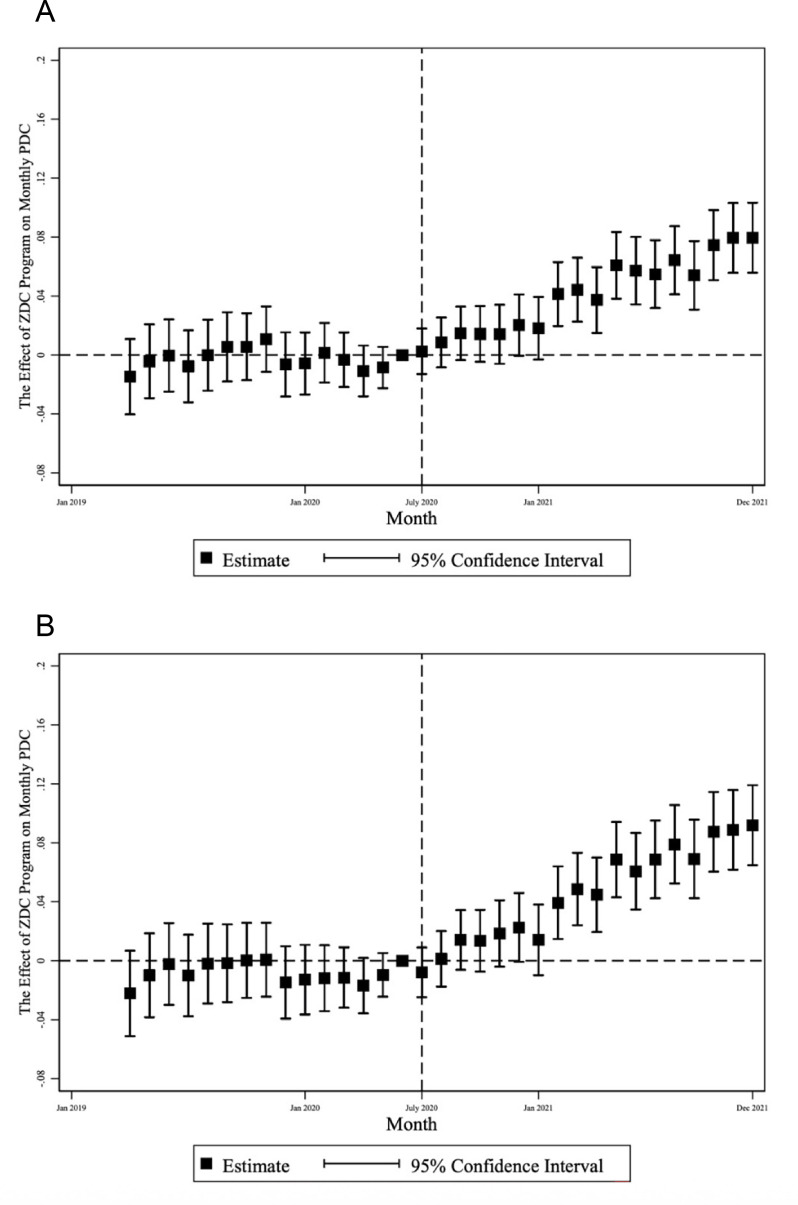
Event studies of effect of ZDC program on monthly PDC, all members, all antidiabetic medications. Note: figure displays leads and lags coefficients of ZDC program effect from difference-in-difference. PDC, proportion of days covered; ZDC, Zero Dollar Copay.

Figure 1, online supplemental appendix figures 1,2, page 2 and 3 show that the preparallel trend assumption was satisfied for all the outcomes of our main analysis. All graphs share similar patterns with no detectable ZDC effects in the baseline period and gradually enlarged statistically significant ZDC effects in the follow-up period.

Table 2 summarizes the treatment effect of the ZDC program for all members and subgroup analyses. For all ZDC members, the ZDC program was associated with a 4.4 (p<0.001) and 6.2 (p<0.001) percentage points increase in PDC and monthly drug use, and a 0.090 (p<0.001) increase in drug counts for members using any antidiabetic medications. For ZDC-eligible antidiabetic medications, the improvements were 5.4 percentage points (p<0.001) for PDC, 7.6 percentage points (p<0.001) for monthly drug use, and 0.074 (p<0.001) for drug counts.

**Table 2:** ZDC program effect on drug utilization

All members	All antidiabetic medications (n=7,603)			ZDC-eligible antidiabetic medications (n=6,419)		
Outcomes	PDC	Drug counts	Any monthly drug use	PDC	Drug counts	Any monthly drug use
ZDC effect	0.044*** (0.0078)	0.090*** (0.012)	0.062*** (0.079)	0.054*** (0.0087)	0.074*** (0.010)	0.076*** (0.0092)
Baseline outcome mean	0.56	0.92	0.64	0.48	0.63	0.56
Relative increase (%)	7.79	9.75	9.59	11.16	11.76	13.53
Annual effect	15.93 days	1.08 drugs	0.74 months	19.71 days	0.89 drugs	0.91 months
**Pre-ZDC users**	**All antidiabetic medications** (**n=3,955**)			**ZDC-eligible antidiabetic medications** (**n=3,955**)		
Outcomes	PDC	Drug counts	Any monthly drug use	PDC	Drug counts	Any monthly drug use
ZDC effect	0.039*** (0.011)	0.053*** (0.015)	0.058*** (0.012)	0.044*** (0.011)	0.063*** (0.013)	0.064*** (0.012)
Baseline outcome mean	0.49	0.62	0.58	0.49	0.62	0.58
Relative increase (%)	7.96	8.46	10.11	9.01	10.05	11.13
Annual effect	14.27 days	0.63 drugs	0.70 months	16.15 days	0.75 drugs	0.77 months
**Pre-ZDC non-users**	**All antidiabetic medications** (**n=1,304**)			**ZDC-eligible antidiabetic medications** (**n=177**)		
Outcomes	PDC	Drug counts	Any monthly drug use	PDC	Drug counts	Monthly drug use
ZDC effect	0.0044 (0.017)	0.025 (0.031)	0.022 (0.016)	0.081 (0.049)	0.020 (0.052)	0.0058 (0.0044)
Baseline outcome mean	0.65	1.10	0.77	0	0	0
Relative increase (%)	0.67	2.23	2.78	NA	NA	NA
Annual effect	1.60 days	0.29 drugs	0.26 months	29.52 days	0.24 drugs	0.070 months
**Complex users**	**All antidiabetic medications** (**n=2,343**)			**ZDC-eligible antidiabetic medications** (**n=2,286**)		
Outcomes	PDC	Drug counts	Any monthly drug use	PDC	Drug counts	Monthly drug use
ZDC effect	0.091*** (0.014)	0.18*** (0.029)	0.10*** (0.014)	0.10*** (0.016)	0.13*** (0.021)	0.13*** (0.017)
Baseline outcome mean	0.64	1.50	0.69	0.52	0.72	0.58
Relative increase (%)	14.21	12.26	14.80	19.38	17.93	22.41
Annual effect	33.31 days	2.21 drugs	1.23 months	36.43 days	1.54 drugs	1.56 months

Table displays estimates of ZDC program effect from difference-in-difference regression. The relative increase percentage was calculated by dividing the estimated ZDC effect by the baseline mean of the outcome in the ZDC group. The annual effect reflects the additional number of days in a year that a member is expected to use medications due to the ZDC pharmacy benefit.

***p≤0.001; no star: p>0.05.

PDC, proportion of days covered; ZDC, Zero Dollar Copay.

For pre-ZDC users who were eligible for the ZDC program, the ZDC program was associated with a 3.9 (p<0.001) and a 5.8 (p<0.001) percentage points increase in PDC and monthly drug use, and a 0.053 (p<0.001) increase in drug counts for members using any antidiabetic medications. For ZDC-eligible antidiabetic medications, the improvements were 4.4 percentage points (p<0.001), 6.4 percentage points (p<0.001), and 0.063 (p<0.001) for PDC, monthly drug use, and drug counts, respectively. However, we did not find significant results for the pre-ZDC non-users.

The largest improvements were observed for complex users who were eligible for the ZDC program. The ZDC program was associated with a 9.1 percentage points (p<0.001) and 10.0 percentage points (p<0.001) increase in PDC and monthly drug use, and a 0.18 (p<0.001) increase in drug counts for members using any antidiabetic medications. For ZDC-eligible antidiabetic medications, the improvements were 10.0 percentage points (p<0.001), 13.0 percentage points (p<0.001), and 0.13 (p<0.001) for PDC, monthly drug use, and drug counts, respectively.

Antidiabetic medication adherence and use patterns were markedly improved for all baseline non-adherent ZDC members (table 3). The ZDC program was associated with a 5.3 percentage points (p<0.001) and 8.5 percentage points (p<0.001) increase in PDC and monthly drug use, and a 0.11 (p<0.001) increase in drug counts for members using any antidiabetic medications. For ZDC-eligible antidiabetic medications, the improvements were 6.0 percentage points (p<0.001), 9.0 percentage points (p<0.001), and 0.97 (p<0.001) for PDC, monthly drug use, and drug counts, respectively. For adherent ZDC members (online supplemental appendix table 1—Appendix page 4–5), the ZDC program was associated with a 3.7 percentage points (p>0.05) increase in PDC, a −0.58 percentage points (p>0.05) increase in monthly drug use, and a 0.026 (p>0.05) increase in drug counts for any antidiabetic medications. For ZDC-eligible medications, the corresponding increases were 0.51 percentage points (p>0.05), –0.058 percentage points (p>0.05), and 0.011 (p>0.05) in PDC, monthly drug use, and drug counts, respectively.

**Table 3:** ZDC program effect on medication adherence and use patterns by baseline adherent groups

All members	Baseline PDC <0.8 (n=4,221)			Baseline PDC ≥0.8 (n=3,382)		
Outcomes	PDC	Drug counts	Any monthly drug use	PDC	Drug counts	Any monthly drug use
ZDC effect	0.053*** (0.012)	0.11*** (0.017)	0.085*** (0.012)	0.0037 (0.0064)	0.026 (0.017)	−0.0058 (0.0060)
Baseline outcome mean	0.329	0.51	0.44	0.947	1.63	0.99
Relative increase (%)	16.04	22.69	19.41	0.40	1.62	−0.58
Annual effect	19.27 days	1.38 drugs	1.02 months	1.37 days	0.32 drugs	−0.07 months
**Pre-ZDC users**	**Baseline PDC <0.8** (**n=2,677**)			**Baseline PDC ≥0.8** (**n=1,278**)		
Outcomes	PDC	Drug counts	Any monthly drug use	PDC	Drug counts	Any monthly drug use
ZDC effect	0.044** (0.015)	0.067 *** (0.019)	0.077 *** (0.016)	0.012 (0.012)	0.016 (0.020)	−0.0060 (0.012)
Baseline outcome mean	0.316	0.43	0.42	0.940	1.11	0.99
Relative increase (%)	14.07	15.58	18.51	1.25	1.45	−0.60
Annual effect	16.20 days	0.80 drugs	0.93 months	4.28 days	0.19 drugs	−0.07 months
**Pre-ZDC non-users**	**Baseline PDC <0.8** (**n=666**)			**Baseline PDC ≥0.8** (**n=638**)		
Outcomes	PDC	Drug counts	Any monthly drug use	PDC	Drug counts	Any monthly drug use
ZDC effect	0.016 (0.028)	0.038 (0.046)	0.044 (0.027)	−0.018 (0.018)	0.023 (0.036)	0.0019 (0.014)
Baseline outcome mean	0.438	0.76	0.61	0.937	1.56	0.99
Relative increase (%)	3.59	5.09	7.30	−1.91	1.45	0.19
Annual effect	5.74 days	0.46 drugs	0.53 months	−6.52 days	0.27 drugs	0.02 months
**Complex users**	**Baseline PDC <0.8** (**n=878**)			**Baseline PDC ≥0.8** (**n=1,465**)		
Outcomes	PDC	Drug counts	Any monthly drug use	PDC	Drug counts	Any monthly drug use
ZDC effect	0.17*** (0.029)	0.35*** (0.049)	0.19*** (0.030)	0.0021 (0.0062)	0.035 (0.034)	−0.010 (0.0051)
Baseline outcome mean	0.271	0.55	0.34	0.963	2.33	1.00
Relative increase (%)	61.28	64.04	56.50	0.22	1.51	−1.03
Annual effect	60.66 days	4.21 drugs	2.31 months	0.77 days	0.42 drugs	−0.12 months

Table displays estimates of ZDC program effect from difference-in-difference regression. The relative increase percentage was calculated by dividing the estimated ZDC effect by the baseline mean of the outcome in the ZDC group. The annual effect reflects the additional number of days in a year that a member is expected to use medications due to the ZDC pharmacy benefit.

***p≤0.001, **p≤0.01; no star: p>0.05.

PDC, proportion of days covered; ZDC, Zero Dollar Copay.

## Discussion

The ZDC pharmacy benefit achieved its policy objective by demonstrating that financial incentives can improve medication adherence, highlighting its potential as a scalable strategy to enhance chronic disease management and promote public health. Antidiabetic medication adherence was significantly improved among the ZDC group, compared with those who did not receive the program. The ZDC group also had increased medication use patterns compared with the non-ZDC group members.

Our study findings of medication adherence are similar to a study that evaluated a pharmacy benefit implemented by Blue Cross Blue Shield of North Carolina. North Carolina’s free medication program was associated with a 3.8 percentage point increase in medication adherence among patients with diabetes.^11^ Similarly, Kim *et al* reported that a reduction in copayments among Medicaid childless adults in Wisconsin can lead to a 2 percentage points increase in PDC of all antidiabetic medications.^12^ Our results are also consistent with the literature that showed correlations between cost-sharing and medication adherence and utilization. For example, for every 10% increase in cost-sharing, there is a decrease of 2–6% in medication use.^13^ Another study reported that doubling the copayments would lead to a 25% reduction in the use of 8 classes of antidiabetic medications.^14^ Moreover, Wang *et al* suggested that a US$5 increase in the medication copayment would lead to a substantial reduction in medication adherence among veterans with diabetes.^15^ On the contrary, the decrease in copayment is usually associated with increased medication adherence.

From a clinical perspective, our findings suggest that the ZDC program may offer meaningful benefits by improving medication adherence, which could translate into better health outcomes. A 12-month randomized clinical trial conducted by Li *et al* reported that adherence in the intervention group exceeded that of the control group by 8%, 7%, and 7% at months 2, 3, and 12, respectively. These improvements were associated with significantly higher probabilities of achieving HbA1c ≤6.5% and total cholesterol ≤5.2 mmol/L.^16^ Similarly, in our study, the ZDC program was associated with a 7.79% relative increase in PDC among all ZDC group members, underscoring the potential of adherence-focused interventions to generate clinically meaningful improvements in chronic disease management. In addition, we evaluated the proportion of members who transitioned from non-adherent to adherent status due to the ZDC effect. Among ZDC group members, the intervention was associated with a 3.3 percentage point higher transition rate compared with the control group (online supplemental appendix table 2, figure 3, page 6, 7). This finding may support the clinical relevance of improved adherence, as prior research found that beneficiaries who were adherent to their medications were nearly twice as likely to achieve control of their disease state compared with those who were non-adherent.^17^

Our study also reports the effect of changes in copayments on monthly medication use patterns, specifically in terms of the number of drugs used per month and overall monthly drug usage. Our findings suggest that the ZDC pharmacy benefit enhances the purchasing power of eligible members and hence enables them to afford more of the antidiabetic medications they need. Previous literature also reported that a reduction in copayment was associated with higher medication utilization. For instance, a study that evaluated a value-based formulary found that moving higher value (estimated by cost-effectiveness analysis) medications to lower copayment tiers would lead to a 1.96 days increase in days of supply in those medications.^12^ Conversely, an increased copayment is usually associated with lower medication utilization. As found in previous research, higher cost-sharing would increase medication termination while decreasing medication initiation and persistence.^18 19^ ^20^

The magnitudes of improvement in PDC and medication use patterns brought by the ZDC pharmacy benefit differ among subgroups. As shown in table 2, the ZDC program had no significant effect on the pre-ZDC non-users subgroup. This finding is reasonable, as the program was auto-enrollment, and individuals who exclusively used non-ZDC medications at baseline would not have experienced any change in their pharmacy costs unless they began using ZDC-eligible medications. Without direct exposure to the program’s financial incentives, these members were likely unaware of the benefit and thus less motivated to change their medication use behavior during the follow-up period. To explain a marked difference in the improvement in the outcomes between pre-ZDC users and complex users, the baseline Diabetes Complication Severity Index (DCSI) scores in the two groups may shed light on the difference (online supplemental appendix tables 3-5, page 8–13). The average baseline DCSI scores in the pre-ZDC group are around 1, while the counterparts in the complex users group are above 2, suggesting that the complex users had at least one more diabetes-related complication on average at baseline compared with the pre-ZDC users. The complex users had a higher need for antidiabetic medications due to worse health profiles, and thus reacted more actively towards the ZDC pharmacy benefit.

For the subgroup analysis of baseline adherent and non-adherent groups, it is possible that non-adherent users were more responsive to financial incentives as the baseline PDCs were 0.39 and 0.33 in the non-ZDC and the ZDC groups among the non-adherent members, respectively (online supplemental appendix table 6, page 14–15).

It is also worth noting that the observed ZDC effect was greater for ZDC-eligible antidiabetic medications within the pre-ZDC users subgroup. Although switching was not assessed in this study, future research could examine potential changes in medication use patterns, including transitions between ZDC-eligible and non-eligible medications.

Our study has several strengths and limitations. First, we conducted our analysis at a monthly level, allowing us to capture variations in outcomes during the follow-up period. This time frame enables us to observe changes promptly and effectively. Second, BCBSLA provided statewide medical and pharmacy claims data, encompassing over 10,000 members with diabetes. This subset represents approximately 2.5% of the total diabetes population in LA, making our study highly representative. Third, the comprehensive and detailed pharmacy claims data allows us to compute monthly drug counts and drug utilization, offering insightful perspectives on medication utilization.

Our most important limitation is that ZDC usage was not randomly assigned, which may lead to unmeasured confounding. It is a strength that ZDC was assigned based on firm characteristics (ASO vs fully insured), rather than allowing individual members to opt into or out of the program. There may, however, be some selection of workers into the types of firms that use ASO. Relatedly, our evaluation is limited to the full implementation of the ZDC program. In prior years, individuals (in fully insured firms) were allowed to select into pilot versions of the ZDC program. These individuals were observed to be sicker and thus had more potential to benefit from the ZDC program. They were also likely more motivated to improve their care. Since we exclude them from our analysis, it is likely to bias our results against the program (we are less likely to detect a positive effect). This bias is a strength of our study, and our results should be interpreted as a lower bound of the program’s effect if it had included these more motivated individuals in the study.

A second limitation is that our patient selection algorithm did not exclude individuals who may have been prescribed certain classes of antidiabetic medications for non-diabetic indications. As such, our results could be interpreted as the impact of financial incentives on the uptake of antidiabetic medications rather than the uptake of antidiabetic medications among patients with diabetic indications. While our study has implications for the health of patients with diabetes, future work will look directly at health outcomes, so we make no strong claims on the impacts of antidiabetic medication adherence on health here.

The generalizability of this study has limits. Our study members were mostly of working age, therefore, we cannot extrapolate our findings to older or younger populations. Additionally, since we did not examine medication use patterns by drug class (ie, oral vs injectables), we were unable to assess whether the ZDC program had differential effects on adherence or utilization across specific types of antidiabetic therapies. Additionally, the PDC was calculated at the antidiabetic class level rather than for individual medications. As a result, it is possible that the PDC for specific antidiabetic drugs was not improved by the ZDC program, even if the overall class-level adherence appeared to increase. This approach may mask variation in adherence across different medications within the same therapeutic class.

In conclusion, the BCBSLA ZDC program was associated with increased medication adherence and medication use among the members with the ZDC pharmacy benefit compared with those without. This study contributes to the literature on copayment adjustments and medication adherence. This highlights the role of value-based benefit design in improving patient access to and continuity of care to ultimately improve health outcomes.

## Supplementary material

10.1136/bmjdrc-2025-005146 Supplementary Figure 1

## Data Availability

Data may be obtained from a third party and are not publicly available.
