# Supplementary material for: Zero Dollar Drug Copay program improves antidiabetic medication adherence and medication use patterns among Blue Cross and Blue Shield of Louisiana members with diabetes in Louisiana
Source: BMJ Open Diabetes Res Care. 2026 Jan 20;14(1):e005146. doi: 10.1136/bmjdrc-2025-005146 (PMC12820829; doi:10.1136/bmjdrc-2025-005146)
Supplement: Supplementary Figure 1 [file bmjdrc-14-1-s001.docx]

***Appendix Contents***

[Figure S1. Event Study for Monthly Drug Count 2](#_Toc200826515)

[Figure S2. Event Study for Monthly Drug Use 3](#_Toc200826516)

[Table S1. The table for the effect of the ZDC Program on drug utilization among members with baseline PDCs ≥ 0.8 4](#_Toc200826517)

[Table S2. The table for the effect of the ZDC program on the proportion of members going from non-adherent group to the adherent group 6](#_Toc200826518)

[Figure S3. Event Study for Adherent Status Transition 7](#_Toc200826519)

[Table S3. Baseline Balancing Table for Pre ZDC Users 8](#_Toc200826520)

[Table S4. Baseline Balancing Table for Pre ZDC Non-Users 10](#_Toc200826521)

[Table S5. Baseline Balancing Table for Complex Users 12](#_Toc200826522)

[Table S6. Baseline Balancing Table for Baseline Non-Adherent Members 14](#_Toc200826523)

[Figure S4. PDC Calculation Algorithm 16](#_Toc200826524)

[Figure S5. Data Exclusion Explanation 17](#_Toc200826525)

[Figure S6. ZDC-Eligible Anti-Diabetic Medications 18](#_Toc200826526)

### Figure S1. Event Study for Monthly Drug Count

Figure S1. Event Studies of Effect of ZDC Program on Monthly Drug Count, All Members


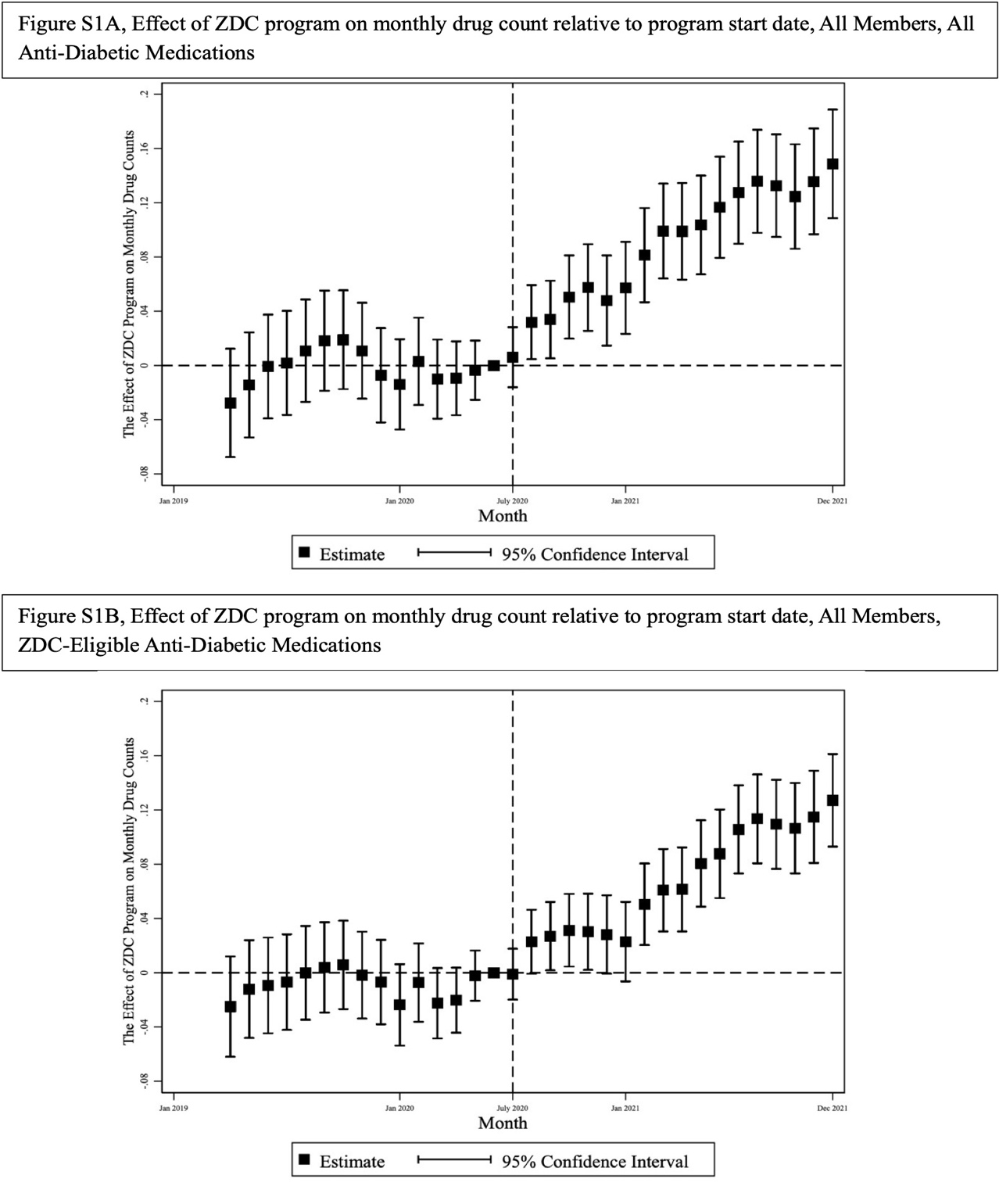


Notes: Figure displays leads and lags coefficients of ZDC program effect from difference-in-difference regression.

### Figure S2. Event Study for Monthly Drug Use

Figure S2. Event Studies of Effect of ZDC Program on Monthly Drug Use, All Members


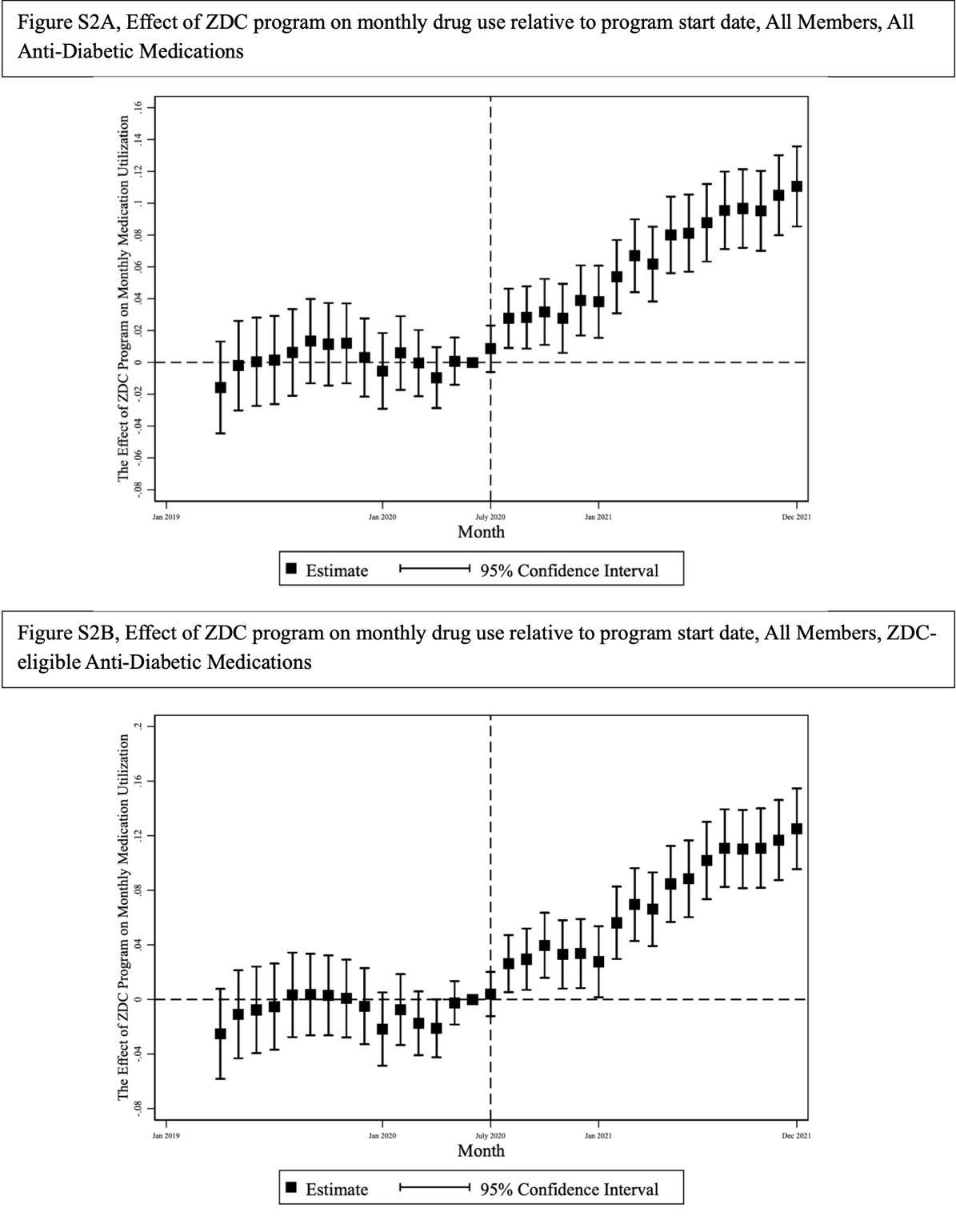


Notes: Figure displays leads and lags coefficients of ZDC program effect from difference-in-difference regression.

| Table S1. The table for the effect of the ZDC Program on drug utilization among members with baseline PDCs ≥ 0.8 **Table S1. ZDC Program Effect on Drug Utilization among Members with Baseline PDC ≥ 0.8** | | | | | | |
| --- | --- | --- | --- | --- | --- | --- |
| **All Members** | **All Antidiabetic Medications**  **(N = 3,382)** | | | **ZDC-Eligible Antidiabetic Medications**  **(N = 2,263)** | | |
| Outcomes | PDC | Drug Counts | Any Monthly Drug Use | PDC | Drug Counts | Any Monthly Drug Use |
| ZDC Effect | 0.0037 (0.0064) | 0.026 (0.017) | -0.0058 (0.0060) | 0.0051 (0.0098) | -0.011 (0.014) | -0.00058 (0.0096) |
| Baseline Outcome Mean | 0.947 | 1.63 | 0.99 | 0.941 | 1.17 | 0.99 |
| Relative Increase (%) | 0.40 | 1.62 | -0.58 | 0.54 | -0.98 | -0.06 |
| Annual Effect | 1.37 days | 0.32 drugs | -0.07 months | 1.86 days | -0.14 drugs | -0.01 months |
|  |  |  |  |  |  |  |
| **Pre-ZDC Users** | **All Antidiabetic Medications**  **(N = 1,278)** | | | **ZDC-Eligible Antidiabetic Medications**  **(N = 1,278)** | | |
| Outcomes | PDC | Drug Counts | Any Monthly Drug Use | PDC | Drug Counts | Any Monthly Drug Use |
| ZDC Effect | 0.012 (0.012) | 0.016 (0.020) | -0.0060 (0.012) | 0.019 (0.013) | 0.019 (0.016) | 0.0063 (0.012) |
| Baseline Outcome Mean | 0.940 | 1.11 | 0.99 | 0.940 | 1.11 | 0.99 |
| Relative Increase (%) | 1.25 | 1.45 | -0.60 | 2.03 | 1.68 | 0.64 |
| Annual Effect | 4.28 days | 0.19 drugs | -0.07 months | 6.97 days | 0.22 drugs | 0.08 months |
|  |  |  |  |  |  |  |
| **Pre-ZDC Non-Users** | **All Antidiabetic Medications**  **(N = 638)** | | | **ZDC-Eligible Antidiabetic Medications**  **(N = 0)** | | |
| Outcomes | PDC | Drug Counts | Any Monthly Drug Use | PDC | Drug Counts | Monthly Drug Use |
| ZDC Effect | -0.018 (0.018) | 0.023 (0.036) | 0.0019 (0.014) | NA | NA | NA |
| Baseline Outcome Mean | 0.937 | 1.56 | 0.99 | NA | NA | NA |
| Relative Increase (%) | -1.91 | 1.45 | 0.19 | NA | NA | NA |
| Annual Effect | -6.52 days | 0.27 drugs | 0.02 months | NA | NA | NA |
|  |  | | |  | | |
| **Complex Users** | **All Antidiabetic Medications**  **(N = 1,465)** | | | **ZDC-Eligible Antidiabetic Medications**  **(N = 978)** | | |
| Outcomes | PDC | Drug Counts | Any Monthly Drug Use | PDC | Drug Counts | Monthly Drug Use |
| ZDC Effect | 0.0021 (0.0062) | 0.035 (0.034) | -0.010 (0.0051) | -0.018 (0.015) | -0.055 (0.029 | -0.0097 (0.015) |
| Baseline Outcome Mean | 0.963 | 2.33 | 1.00 | 0.944 | 1.29 | 0.99 |
| Relative Increase (%) | 0.22 | 1.51 | -1.03 | -1.91 | -4.28 | -0.97 |
| Annual Effect | 0.77 days | 0.42 drugs | -0.12 months | -6.59 days | -0.66 drugs | -0.12 months |
| **Notes**: Table displays estimates of ZDC program effect from difference-in-difference regression. The relative increase percentage was calculated by dividing the estimated ZDC effect by the baseline mean of the outcome in the ZDC group. The annual effect reflects the additional number of days in a year that a member is expected to use medications due to the ZDC pharmacy benefit.  **Abbreviations**: ***: P ≤ 0.001, **: p ≤ 0.01; *: p ≤ 0.05, no star: p > 0.05. | | | | | | |

### Table S2. The table for the effect of the ZDC program on the proportion of members going from non-adherent group to the adherent group

| **Table S2. The Proportion of Members going from Non-Adherent Group to the Adherent Group** | | |
| --- | --- | --- |
| **All Members** | **All Antidiabetic Medications**  **(N = 7,603)** | **ZDC-Eligible Antidiabetic Medications**  **(N = 6,419)** |
| Outcomes | Proportion Transitioning to Adherent Group | Proportion Transitioning to Adherent Group |
| ZDC Effect (%) | 3.33*** (0.78) | 4.05*** (0.85) |
| Baseline Proportion of being Adherent (%) | 49.97 | 43.21 |
| Relative Increase (%) | 6.67 | 9.37 |
|  | | |
| **Pre-ZDC Users** | **All Antidiabetic Medications**  **(N = 3,955)** | **ZDC-Eligible Antidiabetic Medications**  **(N = 3,955)** |
| Outcomes | Proportion Transitioning to Adherent Group | Proportion Transitioning to Adherent Group |
| ZDC Effect | 2.53* (1.08) | 3.14** (1.03) |
| Baseline Proportion of being Adherent (%) | 43.50 | 43.50 |
| Relative Increase (%) | 5.81 | 7.22 |
|  | | |
| **Pre-ZDC Non-Users** | **All Antidiabetic Medications**  **(N = 1,304)** | **ZDC-Eligible Antidiabetic Medications**  **(N = 177)** |
| Outcomes | Proportion Transitioning to Adherent Group | Proportion Transitioning to Adherent Group |
| ZDC Effect | 1.15 (1.92) | 7.44 (5.14) |
| Baseline Proportion of being Adherent (%) | 56.48 | 0 |
| Relative Increase (%) | 2.04 | NA |
|  | | |
| **Complex Users** | **All Antidiabetic Medications**  **(N = 2,343)** | **ZDC-Eligible Antidiabetic Medications**  **(N = 2,286)** |
| Outcomes | Proportion Transitioning to Adherent Group | Proportion Transitioning to Adherent Group |
| ZDC Effect | 8.47*** (1.44) | 9.02*** (1.59) |
| Baseline Proportion of being Adherent (%) | 60.55 | 46.96 |
| Relative Increase (%) | 13.98 | 19.20 |
| **Notes**: Table displays estimates of ZDC program effect from difference-in-difference regression. The relative increase percentage was calculated by dividing the estimated ZDC effect by the baseline mean of the outcome in the ZDC group.  **Abbreviations**: ***: P ≤ 0.001, **: p ≤ 0.01; *: p ≤ 0.05, no star: p > 0.05. | | |

### Figure S3. Event Study for Adherent Status Transition

Figure S3. Event Studies of Effect of ZDC Program on Adherent Status Transition, All Members, All Anti-Diabetic Medications


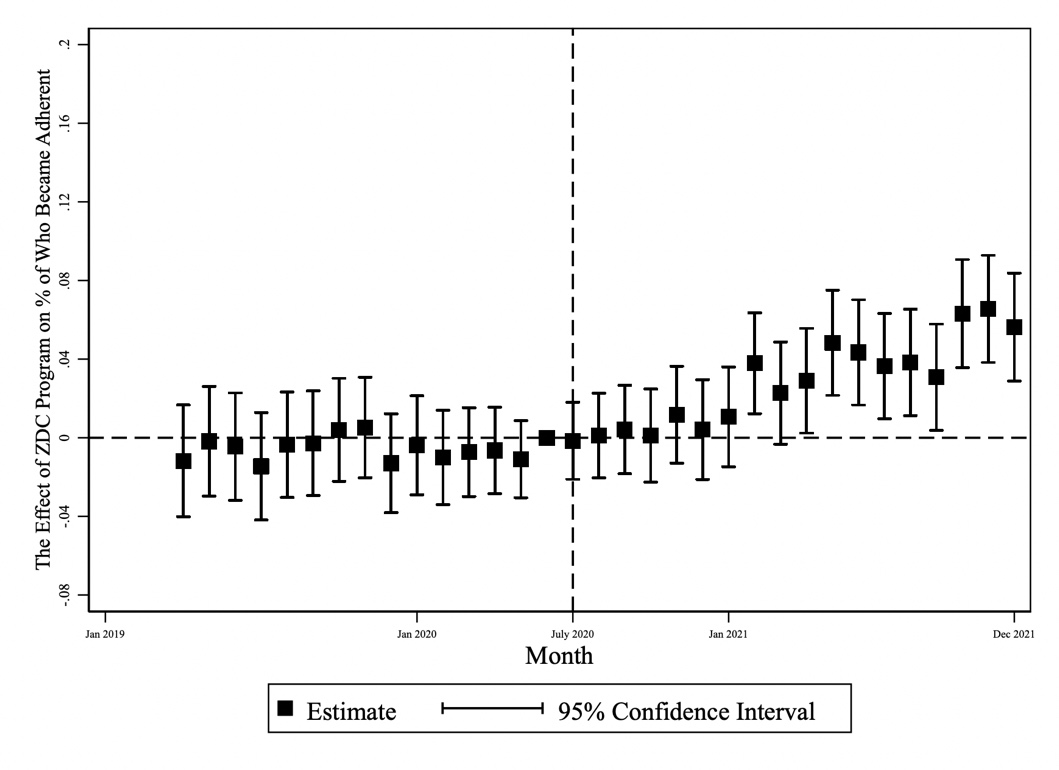


Notes: Figure displays leads and lags coefficients of ZDC program effect from difference-in-difference regression.

### Table S3. Baseline Balancing Table for Pre ZDC Users

Table S3. Baseline Covariates Balance, Pre-ZDC Users, Monthly PDC of All Medications (N = 3,955)

|  | Non-Weighted Group Monthly Average | | | Weighted Group Mean Monthly Average | | |
| --- | --- | --- | --- | --- | --- | --- |
| Variables | Control  (N = 2,205) | Treatment  (N = 1,750) | SMD | Control  (N = 1,758) | Treatment  (N = 1,750) | SMD |
| Age | 51.46 (12.05) | 47.53 (12.95) | -0.31 | 47.45 (12.96) | 47.53 (12.95) | 0.01 |
| Age (≤45) | 633 (28.71%) | 723 (41.31%) | 0.27 | 736 (41.87%) | 723 (41.31%) | -0.01 |
| Age (46 - 64) | 1376 (62.4%) | 927 (52.97%) | -0.19 | 925 (52.59%) | 927 (52.97%) | 0.01 |
| Age (≥65) | 196 (8.89%) | 100 (5.71%) | -0.12 | 97 (5.54%) | 100 (5.71%) | 0.01 |
| Sex (Women) | 1352 (61.32%) | 1158 (66.17%) | 0.10 | 1151 (65.48%) | 1158 (66.17%) | 0.01 |
| Covid | 31 (1.41%) | 28 (1.6%) | 0.02 | 27 (1.53%) | 28 (1.6%) | 0.01 |
| Anxiety | 486 (22.04%) | 382 (21.83%) | -0.01 | 383 (21.76%) | 382 (21.83%) | 0.00 |
| Cancer | 275 (12.47%) | 163 (9.31%) | -0.10 | 157 (8.94%) | 163 (9.31%) | 0.01 |
| CHF | 58 (2.63%) | 14 (0.8%) | -0.14 | 14 (0.78%) | 14 (0.8%) | 0.00 |
| CAD | 198 (8.98%) | 93 (5.31%) | -0.14 | 93 (5.32%) | 93 (5.31%) | 0.00 |
| CKD | 81 (3.67%) | 43 (2.46%) | -0.07 | 44 (2.52%) | 43 (2.46%) | 0.00 |
| COPD | 62 (2.81%) | 23 (1.31%) | -0.11 | 24 (1.38%) | 23 (1.31%) | -0.01 |
| ESRD | 6 (0.27%) | 4 (0.23%) | -0.01 | 5 (0.27%) | 4 (0.23%) | -0.01 |
| Hypertension | 1472 (66.76%) | 750 (42.86%) | -0.49 | 756 (43.03%) | 750 (42.86%) | 0.00 |
| Osteoarthritis | 353 (16.01%) | 205 (11.71%) | -0.12 | 215 (12.24%) | 205 (11.71%) | -0.02 |
| SAD | 84 (3.81%) | 58 (3.31%) | -0.03 | 65 (3.7%) | 58 (3.31%) | -0.02 |
| Urban | 1805 (81.86%) | 1333 (76.17%) | -0.14 | 1336 (75.98%) | 1333 (76.17%) | 0.00 |
| Brand AA | 107.4 (535.18) | 178.1 (914.9) | 0.09 | 253.3 (1283.85) | 178.1 (914.9) | -0.07 |
| Generic AA | 74.25 (227.94) | 59.24 (116.22) | -0.08 | 58.47 (115.13) | 59.24 (116.22) | 0.01 |
| IA | 0.01 (0.03) | 0 (0.02) | -0.07 | 0 (0.02) | 0 (0.02) | -0.01 |
| OP Surgery | 0.04 (0.09) | 0.04 (0.12) | -0.05 | 0.04 (0.08) | 0.04 (0.12) | 0.00 |
| PCP Visit | 0.2 (0.18) | 0.18 (0.18) | -0.15 | 0.17 (0.17) | 0.18 (0.18) | 0.01 |
| SO Visit | 0.54 (0.81) | 0.52 (0.76) | -0.02 | 0.54 (1) | 0.52 (0.76) | -0.02 |
| Office Visit | 0.74 (0.86) | 0.7 (0.8) | -0.05 | 0.72 (1.03) | 0.7 (0.8) | -0.02 |
| UC Visit | 0.04 (0.08) | 0.03 (0.07) | -0.09 | 0.03 (0.07) | 0.03 (0.07) | 0.01 |
| ER Visit | 0.02 (0.05) | 0.02 (0.04) | -0.13 | 0.02 (0.04) | 0.02 (0.04) | 0.01 |
| DCSI Score | 1.18 (2.13) | 0.78 (1.8) | -0.20 | 0.78 (1.8) | 0.78 (1.8) | 0.00 |
| Drug Counts | 0.74 (0.44) | 0.62 (0.43) | -0.27 | 0.63 (0.42) | 0.62 (0.43) | -0.01 |
| Pre-Period Monthly PDC  (Not Included in the Probit Model) | 0.58 (0.34) | 0.49 (0.35) | -0.25 | 0.5 (0.34) | 0.49 (0.35) | -0.01 |

**Abbreviations**: Congestive Heart Failure (CHF), Coronary Artery Disease (CAD), Chronic Kidney Disease (CKD), Chronic Obstructive Pulmonary Disease (COPD), End-stage Renal Disease (ESRD), Substance Abuse Disorder (SAD), Allowed Amount (AA), Inpatient Admission (IA), Outpatient Surgery (OP), Primary Care Physician (PCP), Specialty Office (SO), Urgent Care (UC), Emergency Room (ER), Diabetes Complication Severity Index (DCSI), Proportion of Days Covered (PDC), Standardized Mean Difference (SMD).

**Notes**: Table Displays counts and percentages for categorical variables and means and standard deviations for continuous variables. PDC was not included in the probit regression that predicted treatment status, it is displayed to assessment balance across treatment and control groups.

### Table S4. Baseline Balancing Table for Pre ZDC Non-Users

Table S4. Baseline Covariates Balance, Pre-ZDC Non-Users, Monthly PDC of All Medications (N = 1,304)

|  | Non-Weighted Group Monthly Average | | | Weighted Group Mean Monthly Average | | |
| --- | --- | --- | --- | --- | --- | --- |
| Variables | Control  (N = 721) | Treatment  (N = 583) | SMD | Control  (N = 577) | Treatment  (N = 583) | SMD |
| Age | 52.11 (11.82) | 47.68 (12.67) | -0.36 | 47.71 (13.04) | 47.68 (12.67) | 0.00 |
| Age (≤45) | 179 (24.83%) | 226 (38.77%) | 0.30 | 224 (38.85%) | 226 (38.77%) | 0.00 |
| Age (46 - 64) | 463 (64.22%) | 325 (55.75%) | -0.17 | 320 (55.43%) | 325 (55.75%) | 0.01 |
| Age (≥65) | 79 (10.96%) | 32 (5.49%) | -0.20 | 33 (5.73%) | 32 (5.49%) | -0.01 |
| Sex (Women) | 397 (55.06%) | 266 (45.63%) | -0.19 | 272 (47.06%) | 266 (45.63%) | -0.03 |
| Covid | 14 (1.94%) | 15 (2.57%) | 0.04 | 18 (3.06%) | 15 (2.57%) | -0.03 |
| Anxiety | 145 (20.11%) | 80 (13.72%) | -0.17 | 87 (15.02%) | 80 (13.72%) | -0.04 |
| Cancer | 66 (9.15%) | 45 (7.72%) | -0.05 | 46 (7.92%) | 45 (7.72%) | -0.01 |
| CHF | 39 (5.41%) | 15 (2.57%) | -0.15 | 19 (3.25%) | 15 (2.57%) | -0.04 |
| CAD | 101 (14.01%) | 44 (7.55%) | -0.21 | 45 (7.72%) | 44 (7.55%) | -0.01 |
| CKD | 71 (9.85%) | 34 (5.83%) | -0.15 | 35 (6.02%) | 34 (5.83%) | -0.01 |
| COPD | 29 (4.02%) | 15 (2.57%) | -0.08 | 14 (2.45%) | 15 (2.57%) | 0.01 |
| ESRD | 21 (2.91%) | 5 (0.86%) | -0.15 | 7 (1.23%) | 5 (0.86%) | -0.04 |
| Hypertension | 539 (74.76%) | 279 (47.86%) | -0.57 | 281 (48.74%) | 279 (47.86%) | -0.02 |
| Osteoarthritis | 139 (19.28%) | 61 (10.46%) | -0.25 | 63 (10.96%) | 61 (10.46%) | -0.02 |
| SAD | 36 (4.99%) | 24 (4.12%) | -0.04 | 24 (4.24%) | 24 (4.12%) | -0.01 |
| Urban | 597 (82.8%) | 463 (79.42%) | -0.09 | 462 (80.1%) | 463 (79.42%) | -0.02 |
| Brand AA | 894.66 (1091.73) | 736.3 (1080.67) | -0.15 | 728.54 (915.52) | 736.3 (1080.67) | 0.01 |
| Generic AA | 98.24 (203.93) | 56.96 (112.22) | -0.25 | 61.31 (123.31) | 56.96 (112.22) | -0.04 |
| IA | 0.01 (0.04) | 0.01 (0.03) | -0.19 | 0.01 (0.03) | 0.01 (0.03) | -0.04 |
| OP Surgery | 0.07 (0.13) | 0.04 (0.1) | -0.20 | 0.04 (0.1) | 0.04 (0.1) | -0.01 |
| PCP Visit | 0.24 (0.21) | 0.18 (0.17) | -0.29 | 0.18 (0.18) | 0.18 (0.17) | -0.01 |
| SO Visit | 0.65 (0.85) | 0.56 (0.8) | -0.11 | 0.58 (0.8) | 0.56 (0.8) | -0.03 |
| Office Visit | 0.88 (0.9) | 0.74 (0.83) | -0.17 | 0.76 (0.83) | 0.74 (0.83) | -0.03 |
| UC Visit | 0.04 (0.08) | 0.03 (0.07) | -0.14 | 0.03 (0.06) | 0.03 (0.07) | -0.02 |
| ER Visit | 0.03 (0.07) | 0.02 (0.05) | -0.17 | 0.02 (0.05) | 0.02 (0.05) | 0.01 |
| DCSI Score | 2.86 (3.02) | 1.89 (2.68) | -0.34 | 1.92 (2.69) | 1.89 (2.68) | -0.01 |
| Drug Counts | 1.26 (0.74) | 1.1 (0.72) | -0.21 | 1.1 (0.66) | 1.1 (0.72) | 0.00 |
| Pre-Period Monthly PDC  (Not Included in the Probit Model) | 0.72 (0.3) | 0.65 (0.32) | -0.22 | 0.67 (0.31) | 0.65 (0.32) | -0.05 |

**Abbreviations**: Congestive Heart Failure (CHF), Coronary Artery Disease (CAD), Chronic Kidney Disease (CKD), Chronic Obstructive Pulmonary Disease (COPD), End-stage Renal Disease (ESRD), Substance Abuse Disorder (SAD), Allowed Amount (AA), Inpatient Admission (IA), Outpatient Surgery (OP), Primary Care Physician (PCP), Specialty Office (SO), Urgent Care (UC), Emergency Room (ER), Diabetes Complication Severity Index (DCSI), Proportion of Days Covered (PDC), Standardized Mean Difference (SMD).

**Notes**: Table Displays counts and percentages for categorical variables and means and standard deviations for continuous variables. PDC was not included in the probit regression that predicted treatment status, it is displayed to assessment balance across treatment and control groups.

### Table S5. Baseline Balancing Table for Complex Users

Table S5. Baseline Covariates Balance, Complex Users, Monthly PDC of All Medications (N = 2,343)

|  | Non-Weighted Group Monthly Average | | | Weighted Group Mean Monthly Average | | |
| --- | --- | --- | --- | --- | --- | --- |
| Variables | Control  (N = 1,631) | Treatment  (N = 712) | SMD | Control  (N = 712) | Treatment  (N = 712) | SMD |
| Age | 55.27 (9.41) | 52.75 (9.92) | -0.26 | 52.72 (10.05) | 52.75 (9.92) | 0.00 |
| Age (≤45) | 235 (14.4%) | 154 (21.63%) | 0.19 | 156 (21.87%) | 154 (21.63%) | -0.01 |
| Age (46 - 64) | 1192 (73.1%) | 498 (69.94%) | -0.07 | 494 (69.45%) | 498 (69.94%) | 0.01 |
| Age (≥65) | 204 (12.5%) | 60 (8.43%) | -0.13 | 62 (8.68%) | 60 (8.43%) | -0.01 |
| Sex (Women) | 783 (47.98%) | 323 (45.37%) | -0.05 | 326 (45.85%) | 323 (45.37%) | -0.01 |
| Covid | 15 (0.92%) | 12 (1.69%) | 0.07 | 12 (1.65%) | 12 (1.69%) | 0.00 |
| Anxiety | 307 (18.81%) | 114 (16.01%) | -0.07 | 122 (17.09%) | 114 (16.01%) | -0.03 |
| Cancer | 176 (10.78%) | 76 (10.67%) | 0.00 | 76 (10.67%) | 76 (10.67%) | 0.00 |
| CHF | 80 (4.9%) | 27 (3.79%) | -0.05 | 28 (3.95%) | 27 (3.79%) | -0.01 |
| CAD | 228 (13.97%) | 68 (9.55%) | -0.14 | 69 (9.69%) | 68 (9.55%) | 0.00 |
| CKD | 150 (9.19%) | 46 (6.46%) | -0.10 | 46 (6.51%) | 46 (6.46%) | 0.00 |
| COPD | 62 (3.8%) | 19 (2.67%) | -0.06 | 18 (2.49%) | 19 (2.67%) | 0.01 |
| ESRD | 16 (0.98%) | 4 (0.56%) | -0.05 | 4 (0.58%) | 4 (0.56%) | 0.00 |
| Hypertension | 1357 (83.21%) | 536 (75.28%) | -0.20 | 538 (75.57%) | 536 (75.28%) | -0.01 |
| Osteoarthritis | 278 (17.03%) | 118 (16.57%) | -0.01 | 119 (16.65%) | 118 (16.57%) | 0.00 |
| SAD | 67 (4.11%) | 43 (6.04%) | 0.09 | 42 (5.87%) | 43 (6.04%) | 0.01 |
| Urban | 1283 (78.68%) | 550 (77.25%) | -0.03 | 550 (77.26%) | 550 (77.25%) | 0.00 |
| Brand AA | 746.24 (2399.44) | 568.46 (800.32) | -0.10 | 564.46 (1745.73) | 568.46 (800.32) | 0.00 |
| Generic AA | 99.52 (495.01) | 60.45 (78.52) | -0.11 | 59.83 (74.43) | 60.45 (78.52) | 0.01 |
| IA | 0.01 (0.03) | 0.01 (0.04) | 0.07 | 0.01 (0.05) | 0.01 (0.04) | -0.05 |
| OP Surgery | 0.06 (0.13) | 0.06 (0.13) | -0.02 | 0.06 (0.14) | 0.06 (0.13) | -0.03 |
| PCP Visit | 0.25 (0.23) | 0.21 (0.19) | -0.19 | 0.21 (0.18) | 0.21 (0.19) | -0.01 |
| SO Visit | 0.64 (0.88) | 0.53 (0.77) | -0.13 | 0.55 (0.81) | 0.53 (0.77) | -0.02 |
| Office Visit | 0.89 (0.93) | 0.74 (0.8) | -0.17 | 0.76 (0.84) | 0.74 (0.8) | -0.02 |
| UC Visit | 0.03 (0.07) | 0.03 (0.06) | -0.11 | 0.02 (0.06) | 0.03 (0.06) | 0.01 |
| ER Visit | 0.03 (0.06) | 0.02 (0.06) | -0.05 | 0.02 (0.05) | 0.02 (0.06) | 0.00 |
| DCSI Score | 3.2 (2.92) | 2.63 (2.85) | -0.20 | 2.61 (2.83) | 2.63 (2.85) | 0.01 |
| Drug Counts | 1.88 (1.1) | 1.5 (1.16) | -0.33 | 1.52 (1.06) | 1.5 (1.16) | -0.02 |
| Pre-Period Monthly PDC  (Not Included in the Probit Model) | 0.77 (0.32) | 0.64 (0.4) | -0.35 | 0.67 (0.37) | 0.64 (0.4) | -0.07 |

**Abbreviations**: Congestive Heart Failure (CHF), Coronary Artery Disease (CAD), Chronic Kidney Disease (CKD), Chronic Obstructive Pulmonary Disease (COPD), End-stage Renal Disease (ESRD), Substance Abuse Disorder (SAD), Allowed Amount (AA), Inpatient Admission (IA), Outpatient Surgery (OP), Primary Care Physician (PCP), Specialty Office (SO), Urgent Care (UC), Emergency Room (ER), Diabetes Complication Severity Index (DCSI), Proportion of Days Covered (PDC), Standardized Mean Difference (SMD).

**Notes**: Table Displays counts and percentages for categorical variables and means and standard deviations for continuous variables. PDC was not included in the probit regression that predicted treatment status, it is displayed to assessment balance across treatment and control groups.

### Table S6. Baseline Balancing Table for Baseline Non-Adherent Members

Table S6. Baseline Covariates Balance, All Members with Baseline PDC < 0.8, Monthly PDC of All Medications (N = 4,221)

|  | Non-Weighted Group Monthly Average | | | Weighted Group Mean Monthly Average | | |
| --- | --- | --- | --- | --- | --- | --- |
| Variables | Control  (N = 2,302) | Treatment  (N = 1,919) | SMD | Control  (N = 1,933) | Treatment  (N = 1,919) | SMD |
| Age | 50.17  (11.99) | 46.33  (12.53) | -0.31 | 45.9  (12.9) | 46.33  (12.53) | 0.03 |
| Age (≤45) | 727  (31.58%) | 850  (44.29%) | 0.26 | 893  (46.22%) | 850  (44.29%) | -0.04 |
| Age (46 - 64) | 1402  (60.9%) | 982  (51.17%) | -0.20 | 955  (49.43%) | 982  (51.17%) | 0.03 |
| Age (≥65) | 173  (7.52%) | 87  (4.53%) | -0.13 | 84  (4.36%) | 87  (4.53%) | 0.01 |
| Sex (Women) | 1437  (62.42%) | 1214  (63.26%) | 0.02 | 1218  (63.01%) | 1214  (63.26%) | 0.01 |
| Covid | 36  (1.56%) | 41  (2.14%) | 0.04 | 43  (2.2%) | 41  (2.14%) | 0.00 |
| Anxiety | 510  (22.15%) | 392  (20.43%) | -0.04 | 389  (20.15%) | 392  (20.43%) | 0.01 |
| Cancer | 255  (11.08%) | 163  (8.49%) | -0.09 | 164  (8.48%) | 163  (8.49%) | 0.00 |
| CHF | 78  (3.39%) | 26  (1.35%) | -0.13 | 29  (1.51%) | 26  (1.35%) | -0.01 |
| CAD | 194  (8.43%) | 92  (4.79%) | -0.15 | 93  (4.8%) | 92  (4.79%) | 0.00 |
| CKD | 118  (5.13%) | 50  (2.61%) | -0.13 | 51  (2.62%) | 50  (2.61%) | 0.00 |
| COPD | 72  (3.13%) | 32  (1.67%) | -0.10 | 33  (1.72%) | 32  (1.67%) | 0.00 |
| ESRD | 23  (1%) | 4  (0.21%) | -0.10 | 4  (0.23%) | 4  (0.21%) | 0.00 |
| Hypertension | 1543  (67.03%) | 803  (41.84%) | -0.52 | 802  (41.5%) | 803  (41.84%) | 0.01 |
| Osteoarthritis | 354  (15.38%) | 221  (11.52%) | -0.11 | 225  (11.64%) | 221  (11.52%) | 0.00 |
| SAD | 100  (4.34%) | 77  (4.01%) | -0.02 | 81  (4.19%) | 77  (4.01%) | -0.01 |
| Urban | 1882  (81.75%) | 1451  (75.61%) | -0.15 | 1450  (74.99%) | 1451  (75.61%) | 0.01 |
| Brand AA | 203.67  (627.68) | 238.45  (935.85) | 0.04 | 276.49  (1134.53) | 238.45  (935.85) | -0.04 |
| Generic AA | 61.03  (137.43) | 46.05  (83.56) | -0.13 | 45.5  (82.48) | 46  (83.56) | 0.01 |
| IA | 0.01  (0.04) | 0.01  (0.03) | -0.07 | 0.01  (0.03) | 0.01  (0.03) | -0.03 |
| OP Surgery | 0.05  (0.11) | 0.04  (0.09) | -0.15 | 0.04  (0.09) | 0.04  (0.09) | 0.00 |
| PCP Visit | 0.22  (0.21) | 0.18  (0.19) | -0.19 | 0.18  (0.18) | 0.18  (0.19) | 0.00 |
| SO Visit | 0.54  (0.84) | 0.5  (0.74) | -0.05 | 0.53  (1.12) | 0.5  (0.74) | -0.03 |
| Office Visit | 0.76  (0.89) | 0.68  (0.78) | -0.10 | 0.71  (1.15) | 0.68  (0.78) | -0.03 |
| UC Visit | 0.04  (0.08) | 0.03  (0.07) | -0.06 | 0.03  (0.07) | 0.03  (0.07) | 0.01 |
| ER Visit | 0.03  (0.06) | 0.02  (0.05) | -0.12 | 0.02  (0.05) | 0.02  (0.05) | 0.00 |
| DCSI Score | 1.67  (2.55) | 1.08  (2.16) | -0.25 | 1.09  (2.17) | 1.08  (2.16) | 0.00 |
| Drug Counts | 0.63  (0.49) | 0.51  (0.44) | -0.27 | 0.5  (0.42) | 0.51  (0.44) | 0.01 |
| Pre-Period Monthly PDC  (Not Included in the Probit Model) | 0.39  (0.26) | 0.33  (0.26) | -0.24 | 0.33  (0.25) | 0.33  (0.26) | 0.02 |

**Abbreviations**: Congestive Heart Failure (CHF), Coronary Artery Disease (CAD), Chronic Kidney Disease (CKD), Chronic Obstructive Pulmonary Disease (COPD), End-stage Renal Disease (ESRD), Substance Abuse Disorder (SAD), Allowed Amount (AA), Inpatient Admission (IA), Outpatient Surgery (OP), Primary Care Physician (PCP), Specialty Office (SO), Urgent Care (UC), Emergency Room (ER), Diabetes Complication Severity Index (DCSI), Proportion of Days Covered (PDC), Standardized Mean Difference (SMD).

**Notes**: Table Displays counts and percentages for categorical variables and means and standard deviations for continuous variables. PDC was not included in the probit regression that predicted treatment status, it is displayed to assessment balance across treatment and control groups.

### Figure S4. PDC Calculation Algorithm

Figure S4. PDC Calculation Algorithm


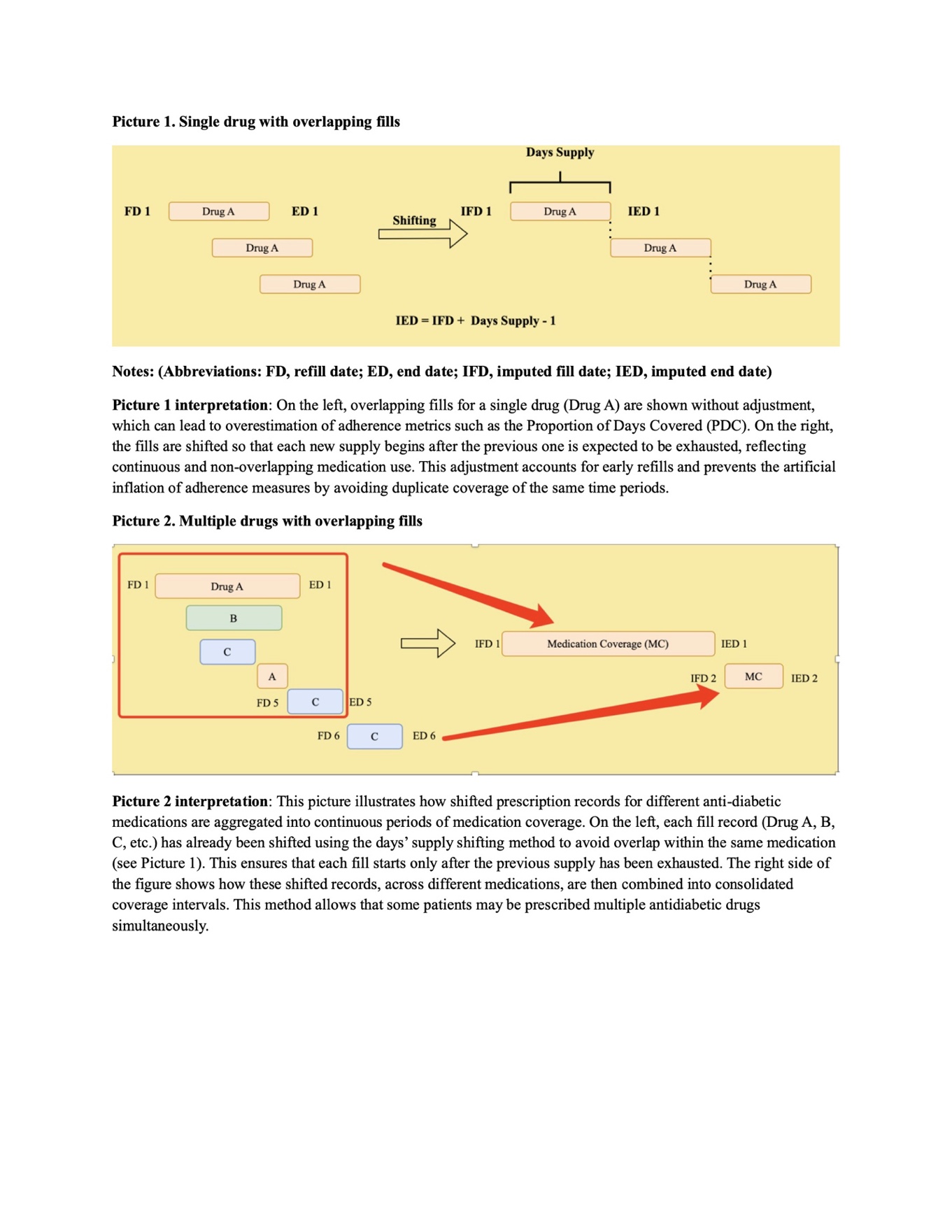


### Figure S5. Data Exclusion Explanation

Figure S5. Data Exclusion Explanation


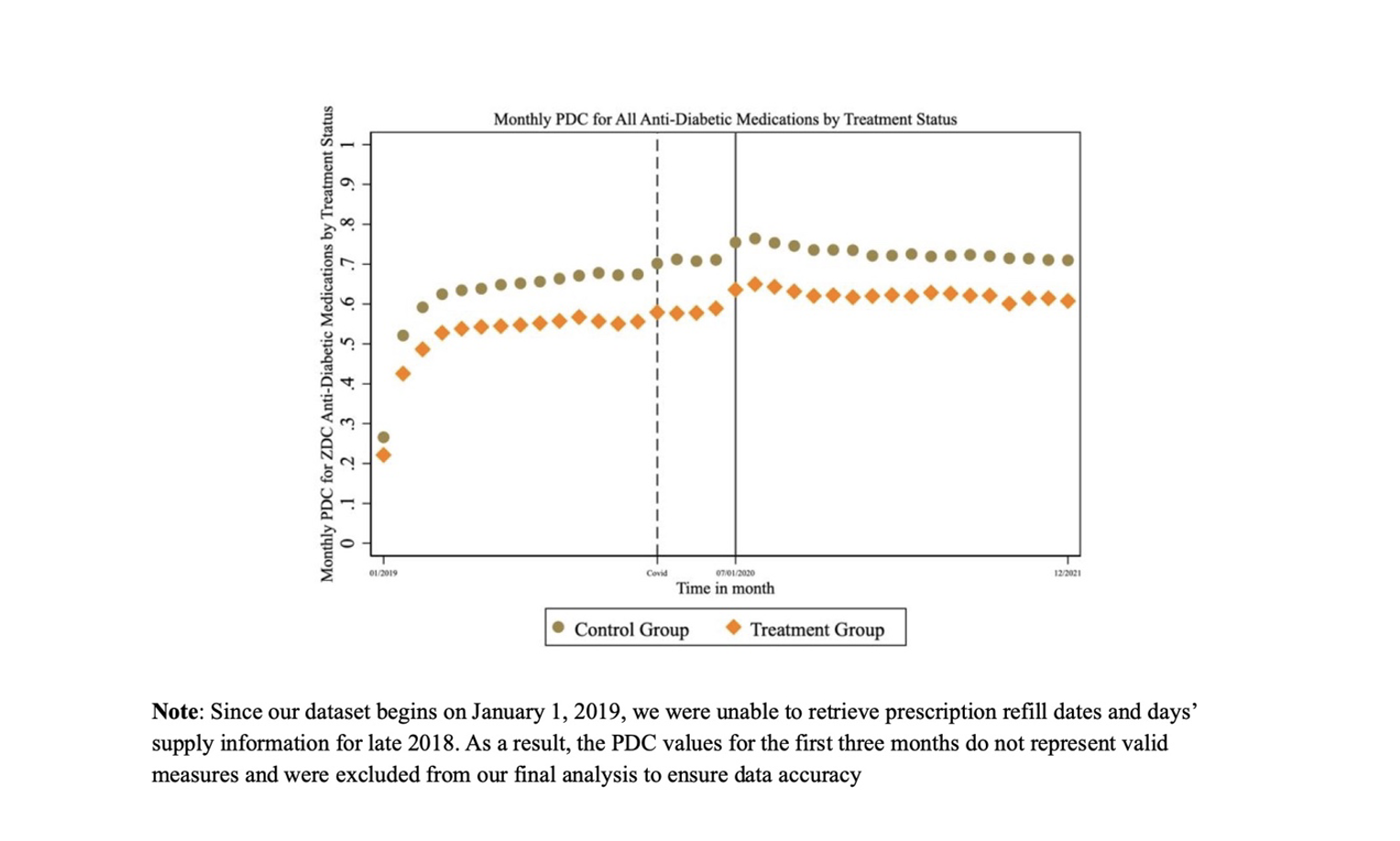


### Figure S6. ZDC-Eligible Anti-Diabetic Medications

Figure S6. ZDC-Eligible Anti-Diabetic Medications


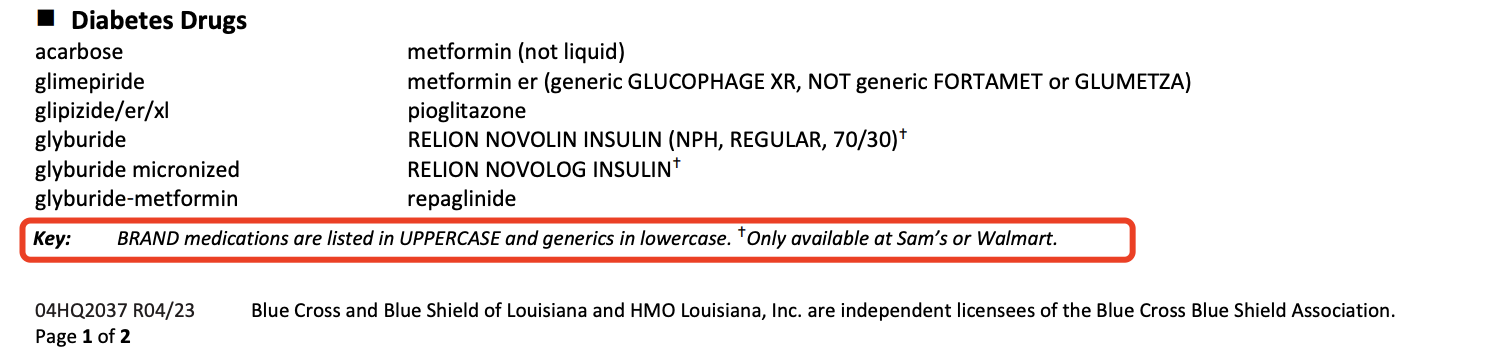


If the audience is interested in the ZDC list for other conditions, please refer to this link: <https://www.bcbsla.com/-/media/Files/Pharmacy/0DrugCopay%20pdf.pdf>
